# Qualitative analysis of national documents on health care services and pharmaceuticals` purchasing challenges: evidence from Iran

**DOI:** 10.1186/s12913-018-3261-0

**Published:** 2018-06-05

**Authors:** Peivand Bastani, Mahnaz Samadbeik, Rassoul Dinarvand, Sara Kashefian-Naeeini, Soudabeh Vatankhah

**Affiliations:** 1 Health Human Resources Research Center, School of Management and Medical Informatics, Shiraz University of Medical Sciences, Shiraz, Iran; 2 Social Determinants of Health Research Center, Lorestan University of Medical Sciences, Khorramabad, Iran; 3 Department of Pharmaceutical Sciences, Faculty of Pharmacy, Tehran University of Medical Sciences, Tehran, Iran; 4 Department of English, Faculty of Paramedical Sciences, Shiraz University of Medical Sciences, Shiraz, Iran; 5 Department of Health Service Management, School of Management and Medical Informatics, Iran University of Medical Sciences, Tehran, Iran

**Keywords:** Qualitative document analysis, Health care services, Pharmaceuticals, Purchasing, Insurance companies, Iran

## Abstract

**Background:**

Iranian health sector encountered many challenges in resource allocation and health service purchasing during the past decades, the aim of this study was to determine the main challenges of the present process of health service purchasing for national policymakers and other developing countries with the same setting.

**Methods:**

It was a qualitative study carried out via the complete content analysis of all relevant national documents from 2007 to 2014. In order to retrieve the related documents, we searched the official websites related to the Ministry of Health and Medical Education, four main Iranian insurance organizations, the Health Committee of the Parliament Profile, strategic vice president’s site and Supreme Insurance Council. After recognition of documents, their credibility and authenticity were evaluated in terms of their publication or adjustment. For the analysis of documents, the four step-Scott method was used applying MAXQDA version 10.

**Results:**

Findings illustrated that health service purchase challenges in the country can be classified in 6 main themes of policy-making, executive, intersectional, natural, legal and informational challenges with 26 subthemes. Furthermore, 5 themes of Basic Benefit Package, Reimbursement,Decision making, Technology and Contract are considered as the main Challenges in pharmaceutical purchasing area containing 13 relevant subthemes.

**Conclusions:**

It seems that according to documents, Iran has faced many structural and procedural problems with the purchase of the best health interventions. So it is highly recommended to consider consequences derived from the present challenges and try to use these evidences in their policy making process to decrease the existed problems and move to better procurement of health interventions.

## Background

Health services purchasing is defined as a process through which the accumulated resources are allocated to healthcare providers by a resource allocation and purchasing (RAP) agency [1]. In other words, purchase is a procurement mechanism of healthcare services whereby accumulated sources are paid to suppliers in order to carry out a set of effective interventions to promote people’s health [2].

Evidences demonstrate that the insurance companies as RAP agencies may purchase healthcare services with two different methods; passive purchase or strategic/active one [3]. Passive purchase is stated to the situation that an insurance company has a passive status in selecting, contracting and procurement of the services and its only duty is reimbursement of the payment for healthcare services which are prescribed for by providers [4]. In contrast, strategic purchasing defines as the constant search for cost-effective, high quality services by an insurance company to procure the best interventions actively. Evidence emphasizes on the vast range of strategic purchase achievements the same as: cost saving, availability, quality and satisfaction of customers and technical and allocative efficiency outcomes [5].

Evidences show that in order to implement strategic purchasing in some developed countries, attempts are made for separating the pooling and purchase functions and also for allocating resources from one pooling organization to purchases via adjusted percapita for obtaining the purchase competition advantages [6]. On the other hand, in these countries, there is an inclination to use models depend on purchase. In these models, governmental and quasi-governmental payers and third party insurers as RAP agencies are organizationally separated from healthcare providers [7]. This can facilitate the potentiality of selecting more effective services and fair-play contracting between the insurer and provider [7]. It is obvious that in the lack of this separation, supplier induced demand may be increased and the power of bargaining, negotiation and purchasing of the insurer will be decreased [4, 8].

Iran as a developing country in the Middle East with about 80 million population has four main governmental social insurance companies. The coverage of insured people by these organizations is totally about 90%. These organizations have similar structure in a way that the mechanism of revenue collection in the term of premium, pooling and purchase are integrated in an organization and resource allocation and purchasing healthcare services are occurred passively through budgeting [9]. In another words, these insurers procure and purchase healthcare interventions for their insurant from both governmental providers affiliated with Ministry of Health and Medical Education and private providers with different tariffs [10]. Despite high insurance coverage in the country, insurance organizations do not have an essential role in the aggregation and management of health service resources. As there is no specific policy in the selection of interventions in resource provision of health service, providers who influence determining policies and resources, play prominent roles in choosing interventions. Continuing this situation, may lead to decrease in resources and lower quality services proceured by insurers and this faulty process will lead to the role of insurance causing a decline in the fair participation in providing financial resources for health services [8].

Correspondingly, various studies have mentioned insufficient insurance coverage, lack of competition due to reliance of most insurance organizations on government, low variety of benefit packages and dissatisfaction of insurant as the most important challenges encountered by insurance organizations in Iran [9, 10]. Rising in insurance organization costs, lack of appropriate referral system, lack of service integrity in different treatment levels, serious problems in payment system to service providers, the purchase of similar services with different tariffs, emphasis on the use of modern technology and expensive ineffective drugs, lack of clarity in the relationships among producers of health services, providers, policy makers, supervisors and service purchasers, attempts of service providers to change implicit needs to explicit demands and high out of pocket payment by the insurant are considered as the other Iranian insurance companies` challenges [2].

As it is obvious although these studies point to some challenges applying experts opinions, their results need to become more complete and applicable by an exact attention to existing policies, regulations and programs and the contrasts between these documents and the experts points of view. For instance, high-level legal requirements of the country including the fifth social-economical development laws requires the health insurance to purchase strategically health services from governmental and non-governmental sections considering referral system and ranking national services and approved policies and priorities of health system with the aim to achieve its pervasive and fair coverage, to increase the availability of health services and to purchase the highest quality and most effective services [11].

According to what was said, the present study deals with the complete analysis of all relevant national resources to determine challenges of the present process of health service purchasing for national policymakers and those with the similar problems like Iran. The contribution to knowledge for the present study is to complete the previous studies by determining the main challenges in purchasing health care services and pharmaceuticals in Iranian health sector from the perspective of national documents, laws and legislations other than experts opinions that was considered before.

## Methods

### Design

The current study is a qualitative one which is carried out via the qualitative narrative document analysis. There are various methods for collecting qualitative data. Gupta believes that in performing qualitative studies, one can use some of the previous data such as documents or other textual data. By existing textual data and documents in this type of research, instructions and governmental circulars such as official documents, programs and issued policies and periodic reports are meant [12]. At the same time Krippendrof believes that documents or other text data like government guidelines and directives, official documents, programs and policies and periodic reports can be analyzed in a hermeneutic approach through a five-step process containing access to documents and data, checking the validity of documents, comprehending the documents, analyzing the data and applying the information in the form of extracted themes [13]. In this approach, the qualitative researcher aims to seek those themes existing in one document or the groups of documents that requires an exact investigation of the documents in order to comprehend the viewpoints of the writers or experts who prepared those texts [14].

As the national documents, laws and legislations can be considered as the main policy-making guidelines, and their contents can clarify the main viewpoints and strategies of that country in each scope, in order to answer the present research question to achieve the main challenges in purchasing health care services and pharmaceuticals in Iranian health sector, the qualitative document analysis was applied as the study methodology.

### Data collection

In this regard, as we thought that formal laws and legislations accompanied with other formal documents and reports existing in health care sector may lead us to many present challenges in health care purchasing, we decided to retrieve the related documents systematically, we searched the official websites related to the Ministry of Health and Medical Education [http://www.behdasht.gov.ir/], Social Security Organization [http://www.msio.org.ir], Iran Health Insurance Organization [http://tamin.ir], Imam Khomeini Relief Committee [http://ihio.gov.ir/], Armed Forces Health Care Insurance[http://mod.ir/], as four main insurance organization in Iran, the Health Committee of the Parliament Profile [http://rc.majlis.ir/fa/parliament_commission/health], strategic vice president’s site (the Management and Planning Organization) [https://www.mporg.ir] and Supreme Insurance Council [http://centinsur.ir/].

In order to create a logical trend and to prevent the loss of relevant data, the document collection and analysis form was used. This form was prepared by the research team for the initial investigation of existing documents and records. The items including this form were the name of the document, the place of retrieving the document and related clarifications about the content of the document in the above scopes. The content validity of the data collection form was confirmed by the research team.

In order to collect data, first we tried to search the above websites electronically applying related keywords and phrases regarding “financial provision methods of health services”, “health insurance”, “the purchase of health services” and “the present situation of purchasing health services” and “pharmaceutical purchasing” from 2007 to 2014. As we thought some documents could not be retrieved electronically, one of the researchers referred to library resources and centers of the Ministry of Health and Medical Education, Food and Drug Organization, the Research Center of the Parliament and basic insurance companies of the country. This set of documents were chosen because the stated organizations and their related documents, laws and legislations contain all the Iranian legal considerations in health sector and purchasing health care services as well and they can be used as a comprehensive source of documents leading us to answer the research question.

### Validation method

**Fig. 1:**
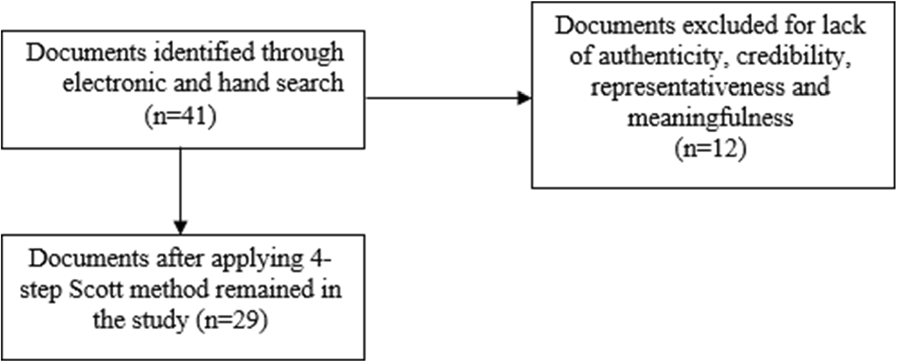
The chart of document acquisition

A 4- step Scott method was used for data collection in order to determine authenticity, credibility, representativeness and meaningfulness of data [15]; in the first step in order to assure authenticity, the offering source of documents was investigated in a way that in this stage those documents which were verified by the Parliament, government, Supreme Insurance Council and insurance organizations were regarded authentic.In the second step as a process of determining credibility, only those documents were verified which were not misleading and were free of mistakes and in general did not have any personal and organizational conflict of interest. Representativeness in the third stage meant that the investigated documents showed general policies or determined keywords on the basis of purpose of research and finally at the last step, meaningfulness meant that the document was clear and comprehensive and in a way had face and content validity. In other words, both its appearance and format should have credibility. Identified documents, after quadruple stages according to Scott criterion were subjected to content analysis in the next stage word by word explicitly and implicitly in an inductive and deductive way [16]and those documents which do not have even one of the quadruple indexes including by Scott were excluded from content analysis. In this regard a total of 29 documents out of 41 included in the study in nine main categories consisting national plans, laws, regulations, policies and legislations (Table 1). Finally, these included documents contain general present challenges of Iranian context and the proposed policies, laws and regulations for improving the situation as well (Fig. 1).

**Table 1:** Final categories of studied documents

No	Final categories of studied documents	Number of documents
1	Fourth social economic development plan	1
2	Fifth social economic development plan	1
3	Health care purchasing executive regulation	4
4	Documents of superior council of insurance	7
5	Regulations according to basic benefit package	4
6	Regulations according to pharmaceutical inclusion criteria and pharmacopeias	4
7	Regulations about health technology assessment	3
8	Regulations about health tariffs	2
9	Laws and regulations related with supplementary insurers	3

### Data analysis

Content analysis dealt with both deductive and inductive approaches for data analysis. The description of analysis was as follows:

In the first phase, we chose a deductive approach to verify the main categories of purchasing challenges in health system. The predetermined codes for categorising the challenges were extracted through the literature review. These codes contain pharmaceutical purchasing challenges and health care purchasing challenges. In this regard, explicit analysis was applied. We looked for the exact words or phrases containing all of the followings: “challenges OR problems OR difficulties”, “purchase OR purchasing OR procure OR procurement”, “health OR health system”. After finding the exact keywords, we highlifht them in the whole body and only went through the frequency and repetition of the words [17]. By considering the more repeated words, we confirmed the main deterrmined codes in this phase.

In the secend phase, the related items were attributed to one of the main categories using inductive approach applying a 5-step framework. For the inductive analysis, first the authors read all the texts for several times to familiarize with the content, then at the next step, we tried to develop a framework assisting the pre determined key words and expressions and during this step all the texts were reviewed again in order to highlight the meaningful units of the text with an implicit approach, at the same time in this step we tried to agree on the meaning definition of “challenge” in health care purchacing and pharmaceuticals as well. In this regard, we searched the concepts and similar topics such as comprehensive health coverage, Health insurance, health services purchase, financial provision of health, etc. in the documents` content, then at the third step, we started to initial indexing and creating codes in a way that after finding each of the above phrases or words based on the researchers critical assessment and their privious knowledge as an expert in this scope (P.B wrote a dissertation in healthcare and pharmaceutical purchasing and R.D is a policy maker in healthcare scope), we highlighted them in the body and then devoted an appropriate code for each determined pharase. These codes were reviewed for many times to develop new codes and be assured of merging the repeated ones, MAXQDA version 10 was used in this step. Then at the fourth stage charting was occurred and we tried to merge the related codes to generate sub categories as sub themes and then achieved to main categories or themes. In this step the relation between the main and subthemes were identified too and at the last step, all the themes and subthemes were interpreted and approved by the research team that did not have any conflict of interest [18].

About the ethical consideration of the study it is important to consider that all the documents and their content were analyzed in the way that there was no name of any of Iranian politicians, policy makers or executive managers, and only their content was used for a qualitative analysis without any conflict of interest by the research team.

## Results

The findings of the obtained document analysis indicated that in the insurance system of Iran the purchase of health services is done passively. In other words, summing up obtained data driven from present condition of the purchase of health services demonstrated the fact that what has been done by insurance organizations regarding the identification of the best providers/suppliers of services follows traditional supervision programs without any potentiality for ranking the best suppliers by the insurance organization and also no power for the organization to bargain, negotiate and contract with the best selected supplier and at present, it seems from evidences that a comprehensive program for ranking healthcare providers has been missing yet. Furthermore, there is no possibility of the complete identification of current novel medical services, the purchase of best services is not carried out. In addition, the most widespread method which is a criterion in insurance organizations payment system, is that of payment in return for service reception. Finally, only the villagers and nomads insurance fund (it is a part of Iran Health Insurance Organization) use a mass purchase mechanism for procurement of services and the other insurance organization just reimburse the payments related to the prescriptions that occurred retrospectively.

However, obtained data from insurance organizations denote their contract formation with more than 45,000 providers of diagnostic and curative services in 2013. These providers spread out all over the country and Ministry of Health and Medical education suffers an inefficient system for their evaluation and supervision because of the lack of applied policies, inappropriate mechanism for monitoring them and concentrating the duties of policy making, implementing and evaluating in the same units.

Other obtained data which are taken from investigated document analysis illustrated that health service purchase challenges in the country can be classified in six main themes of policy-making, executive, intersectional, natural, legal and informational challenges. In this regard Table 2 depicts the most significant themes (6) and sub-themes (26) which are gained from present document analysis.

**Table 2:** Health Care Services Purchasing challenges of Iranian Insurance Companies

Themes	Sub Themes
Policy making Challenges	External and political forces on insurers for contracting with providers
	ambiguities in strategic purchase policies
	lack of regulations, evaluation instructions and ranking providers of services
	Lack of purposefulness of subsidies for purchasing agencies
	lower per capita income in those under medical services coverage
	High rate of out of pocket because of low coverage of services
	great number of households facing catastrophic costs especially in outpatient services
Executive Challenges	lack of effective referral system based on family physician in the whole country
	different prices for the same service by insurance organizations
	heterogeneous distribution of medical experts in different parts of the country
	lack of realization of universal health coverage
	lack of exclusive use of power of purchase (use of inactive purchase).
Intersectional Challenges	Low level of relations between insurance organizations and scientific& expert associations
	lack of competition among insurance organizations
	lack of clarity in the relationships among producers of health services, providers, policy-makers, supervisor and purchaser of services in the country
	the existence of supplementary insurances which buy basic services several times that of the original price
Natural Challenges	The existence of high induced demand in outpatient and drug sections
	Existence of spiral of death because of adverse selection outbreak
	Radar syndrome because of the lack of continuity in offering services level
	Cream scheming in some insurance companies
Legal Challenges	Lack of an exact and scientific basis in assessing insurance rights and tariffs
	The high range of inpatient in patient costs
	Persist on implementing the governmental model of family physician and referral system
	lack of regulations and instructions of health care delivery
Informational Challenges	lack of a comprehensive information bank
	lack of sufficient familiarity with successful health systems experiences in strategic purchase of health services

Though the aforementioned challenges are generally offered by insurance organizations in the country for the purchase of all health services, the coverage of these drawbacks in pharmaceutical section is evidently one of the most important health services. To put it another way, all the six mentioned challenges can be verified in the theme of purchase and preparation of pharmaceuticals. Moreover, other problems other than the above challenges were identified by insurance organizations which are put in the forms of five themes and thirteen sub-themes in Table 3.

**Table 3:** Pharmaceutical Purchasing challenges of Iranian Insurance Companies

Themes	Sub Themes
Basic Benefit Package Challenges	Narrow pharmaceutical packages
	Different pharmaceutical packages among various insurers
Reimbursement Challenges	Incorrect cycle of funds in pharmaceutical section by theinsurers
	Incorrect and long process of pharmaceuticalreimbursement
	Difference in payment ceilings for the same pharmaceuticals
	Difference in payment levels and payment mechanisms of pharmaceutical by various insurers
Decision making Challenges	Existence of adverse selection and moral hazard in pharmaceutical sector
	Lack of attention to cost effectiveness implications in pharmaceutical purchasing
Technology Challenges	Force on applying new technologies and expensive pharmaceuticals
	Devoting great costs to pharmaceutical sector and high technologies
Contract Challenges	Lack of existence fair contracts for pharmaceutical purchasing
	Lack of purchasing grand use pharmaceuticals through mass purchase
	Lack of ranking the pharmaceutical providers by policy makers

As it is depicted in Table 3, insurance organizations do not follow the same pharmacopeia for the purchase of medicines. In other words, each insurance organization has a different basic drug list. However, most people who are insured by basic insurance funds especially the Health Insurance fund have scant availability to needed drug and does not suffice the pharmaceutical requirements of the poor and vulnerable people.

On the other hand, other basic insurance organizations of the country do not pay sufficient attention to moral hazard and adverse selection leads to the expenditure of a bulk of medicinal credit resources for only a few limited people who unfairly cause the highest medicinal costs. In addition, at the time of allocation of resources to different drug categories and drug items cost-effectiveness and economical evaluation are not much considered in decision-making.

The inappropriate and long reimbursement procedure of drug costs to patients by insurance organization _only after paying out of pocket for a large spectrum of expensive drug by patients having refractory diseases _ is an important challenge which is put in Table 3. This improper drug reimbursement together with different payment levels and limits among insurance organizations for the same services is another challenge of this theme.

Exerting pressure by providers of services and pharmaceuticals is another principal challenge of this theme, in another words, because of the lack of accepted official guidelines, providers like physicians have the power to prescribe those pharmaceuticals and services that they prefer.

because of the brand loyalty or sometimes their personal benefits but without any confirmed cost-effectiveness indexes or approved evidences. Moreover, a great amount of costs of basic insurance organizations is spent on pharmaceuticals and medical equipment. It can be a problem when we consider that a great portion of this amount is allocated to antimicrobial medicines. Some of them are prescribed with no indications the same as those are used as prophylaxis and those prescribed irrationally. Biopharmaceuticals and OTCs that are usually considered as brand medicines are the other costing center in this area. In this regard, according to existing evidences, the highest rank is related to Oil Company as one of the minor insurance companies in the country and Social Security Organization and the lowest is pertinent to supplementary insurances. Supplementary insurances are created because of the weak coverage of the basic packages of the social insurance companies in the country. They are considered as private purchasers with higher premium and only rich people with the higher potentiality of payment can use their coverage especially for expensive treatments and medicines.

Finally, lack of ranking of pharmaceutical supplies by supervisional institutions such as Ministry of Health and Medical Education is among other challenges which cause some problems in forming appropriate contracts which are agreed on and the purchase and preparation of most effective drug for insurance organizations. This challenge causes that a purchaser could not have the power of negotiation, bargaining and contracting with the best approved supplier of pharmaceuticals.

## Discussion

Implementing healthcare strategic purchasing especially for developing countries is emphasized by World Health Organization and the World Bank [19]. The present findings indicate some challenges in the way of achieving strategic purchasing in Iranian health sector as: Policy-making, executive, intersectional, natural, legal and informational challenges.

In policy-making, the current findings emphasize the ambiguities in strategic purchase policies and lack of regulations, evaluation instructions and ranking suppliers and providers. In this respect, Moghaddasi et al. considered the lack of clarity of insurance policies in procuring medical service as the most significant obstacle for optimal purchase of health services [16]. Maleki et al. also regards policy-making in determining insurance rights as one of the chief drawbacks in Iran insurance organizations [20]. Lack of purposefulness of subsidies for purchasing agencies are among other present policy making challenges. Likewise, Maleki referred to lack of purposefulness of subsidies [21]. It seems that giving subsidies to purchasing institution can lead to strategic purchasing and also maximizing the access and equity than offering subsidies to providers or patients. Some more challenges in policy-making consider those under medical services coverage have low per capita income in comparison with others. These people face more out of pocket payment because of low coverage of services and great number of households facing catastrophic costs especially in outpatient services. In this regard, lack of a system for putting tariffs, unreal per capita income in the country, unfair and retrogressive financial provision system are among the challenges mentioned by Dehnaviehet al [22]. VafaeeNajjar et al. has referred to mere coverage of expensive admission and recognition services with a specific limit for cost payment by these organizations [23]. This can justify the current findings on catastrophic costs in outpatient section.

The most executive challenges are indicated as: lack of effective referral system based on family physician, different prices for the same service by insurance organizations, heterogeneous distribution of medical experts in different parts of the country, lack of universal health coverage and lack of exclusive use of power of purchase. In this regard, Hassanzadeh, has mentioned that the demand of the patients and induced demand toward suppliers are determinant factors for purchasing services instead of the community real needs and evidence-based guidelines [2]. Moreover, lack of appropriate referral mechanism is another challenges in insurance system in the country [2]. Although from the year 2005 the rural family physician proposal for all villages and from 2011 the urban family physician plan were piloted for two provinces as a referral system, evidences show that not much success has been achieved in solving executive problems of insurance organizations [11]. So, it seems that if Islamic republic of Iran plans to reach the goal of universal health coverage as a serious executive solution, it needs a systematic approach which is based on planning. In a corresponding way, Young et al. have considered essential the four stages of determining interventions, selecting interventions, evaluating technology and evaluating interventions as an optimal strategy for determining interventions which are to be placed in the service package and access to a universal health-coverage [24].

Intersectional challenges are devided to low level of relations between insurance organizations and scientific associations, lack of competition among insurance organizations, lack of clarity in the relationships among producers of health services, providers, policy-makers, supervisor and purchaser in the country and the existence of expensive supplementary insurances. Correspondingly, Hassanzadeh pointed to the lack of clarity in the relationships among producers, providers, policy-makers, supervisors and purchasers of services [2] which similar to the findings of the current study underlines the necessity of intersectional and intra-sectional interactions and creation of a correct supervision mechanism and exact definition of policy-making cycle.

The most important natural challenges include the existence of high induced demand in outpatient and drug sections, adverse selection, radar syndrome (lack of following people by a referral system and just providing services at the time of illness), lack of continuity in offering services and cream scheming in funds. In this respect, Dehnavieh has referred to the high levels of induced demand [18]and Hassanzadeh has mentioned the openness of medical services` supply through an increase in the number of providers due to the training of extra labor force and the occurrence of induced demand and increased expenses in insurance organizations and out of pocket payment [2].

Among legal challenges, the present findings declare the lack of an exact and scientific basis in assessing premiums and tariffs. In this regard, lack of scientific premiums, the retrogressive state of premiums, unreal tariffs and under the table health fees were the most important drawbacks identified by Maleki et al. [21]

Finally, the current informational challenges were the lack of a comprehensive information bank and lack of sufficient familiarity with successful health systems experiences in strategic purchase of health services. It seems that an emphasis on compilation and application of standard instructions, distribution of evaluation system of technology and creation of a comprehensive information system can help solving these challenges.

Regarding pharmaceutical purchase challenges the present findings particularly demonstrate the challenges related to purchase basic benefit package, reimbursement, decision-making, technology and contract.

An important challenge is that pharmaceutical packages do not have enough coverage and there are different pharmacopeias approved by different insurance funds. In this respect, offering national drug pharmacopeia seems essential**.** Highlighting cost-effectiveness requirements are among other aspects which should be considered as an important factor while purchasing and as an important criterion while compiling, changing and amending service package by insurance organizations though the present findings mention it as a decision-making challenge. Correspondingly, Brenfort et al. indicated that for including pharmaceuticals to Sweden basic service package, need criterion was only taken into consideration and cost-effectiveness criterion was rarely regarded [25]. However, Makundi et al. emphasized the revision of evidences related to selected cost-effectiveness of interventions through systematic search of Medline and Cockrane on cost-effectiveness of interventions [26].

Other findings lay emphasis on putting pressure for the use of modern technology and expensive drug. In this regard, Hassanzadeh has acknowledged that a push for the use of modern technology and expensive, ineffective drug along with uplifting expectations of the society using unusual comparisons can impose a lot of trouble on insurance organizations [2].

The lack of possibility to make a win-win contract between purchasing organizations and drug suppliers and the lack of possibility to choose the best supplier from insurance organization due to lack of ranking by the Ministry of Health and Food and Drug Organization are among other challenges in the present study which can pose great problems in putting strategic purchase into practice, in this regard, Arney et al. claimed that applying the strategic plan for contracting can provide the opportunity for sustainable improvements inprocurement efficiency and commodity availability [27]at the same time other evidences showed that using strategic purchasing in pharmaceutical sector can not achieved without improving the ability of contracting between purchasers and suppliers [16].Bastani et al., also emphasized that pharmaceuticals price, real and fair premiums along with the ability of fair contracting with the main suppliers and correcting reimbursement mechanisms toward purchasers are considered as contracting and purchasing incentives [28].

From the main limitations of the study we can point to the lack of complete access to all libraries specially those related to Armed Forces Health Care because of their confidentiality, another limitation can be the study perspective that focused on formal documents, laws and legislations.

## Conclusion

With respect to what has been stated, it seems that according to documents, Iran has faced many structural and procedural problems in the purchase of the best health interventions, particularly in the purchase of drug. It is crystal clear that only by highlighting present laws and regulations and offering strategies for applying them along with designing appropriate organization structure, reducing concentration in decision-making, creating information integration, intensifying competition in health services purchase market and establishing optimal system for the evaluation of both institutions offering services and purchasers, the country can reduce the present challenges and move towards strategic purchase as a strategy for increasing justice and efficiency.
